# 

**Published:** 1907-10-05

**Authors:** 


CONTENTS.

| | PAGE |
|---|---|
| **Professional Freedom** | 1 |
| **A Supreme National Health Authority** | 2 |
| **Annotations—** | |
| Morphine and Retention of Urine—Lectures on Old Age—More Poisonous "Tabloids" | 3 |
| **Medical Opinion and Movement** | 4 |
| **Hospital Clinics—** | |
| How to Perform a Post-mortem Examination: The Experience of a Lifetime, and its Practical Teaching. By Sir Henry D. Littlejohn, M.D., LL.D. (Edin.) | 5 |
| **Practical Notes on Diagnosis and Treatment** | 8 |
| **Illustrative Cases—** | |
| Infarction and Rupture of the Heart | 9 |
| **Points in Diagnosis—** | |
| Paratyphoid Fever | 10 |
| **Points in Surgery—** | |
| The Treatment of Fractures from a Common-sense Point of View | 11 |
| **Diseases of Children—** | |
| Erythema Nodosum | 13 |
| **Poor-law Department—** | |
| The Poor-law Infirmary | 14 |
| **Public Health and Hygiene—** | |
| The Hygiene of the Public Telephone | 15 |
| Race Progress and the Declining Birth-rate | 15 |
| **Points in Pathology—** | |
| Laboratory Methods | 16 |
| **Resident Medical Officers' Department—** | |
| The Habits of the Medical Student Past and Present | 17 |
| **Therapeutics—** | |
| Prescriptions and Dispensing | 18 |
| **Hospital Administration—** | |
| The Bristol Royal Infirmary Impasse | 19 |
| The Administration of Charity | 20 |
| Sane Persons in Asylums | 21 |
| News and Coming Events | 22 |
| New Infirmary for the Wharfedale Union at Otley, Yorkshire | 23 |
| New Appliances for Hospital Use | 24 |
| **Nursing Administration—** | |
| The Nursing Institute | 25 |
| The Common Task | 26 |

